# Evaluating the Work Productivity of Assembling Reinforcement through the Objects Detected by Deep Learning

**DOI:** 10.3390/s21165598

**Published:** 2021-08-19

**Authors:** Jiaqi Li, Xuefeng Zhao, Guangyi Zhou, Mingyuan Zhang, Dongfang Li, Yaochen Zhou

**Affiliations:** 1 Faculty of Infrastructure Engineering, Dalian University of Technology, Dalian 116024, China; lijq@mail.dlut.edu.cn (J.L.); zgy0829@163.com (G.Z.); myzhang@dlut.edu.cn (M.Z.); lidongfang0303@163.com (D.L.); 2 State Key Laboratory of Coastal and Offshore Engineering, Dalian University of Technology, Dalian 116024, China; 3 Northeast Branch China Construction Eighth Engineering Division Corp., Ltd., Dalian 116019, China; zhouyaochen1949@163.com

**Keywords:** construction engineering, construction management, work productivity, computer vision, deep learning

## Abstract

With the rapid development of deep learning, computer vision has assisted in solving a variety of problems in engineering construction. However, very few computer vision-based approaches have been proposed on work productivity’s evaluation. Therefore, taking a super high-rise project as a research case, using the detected object information obtained by a deep learning algorithm, a computer vision-based method for evaluating the productivity of assembling reinforcement is proposed. Firstly, a detector that can accurately distinguish various entities related to assembling reinforcement based on CenterNet is established. DLA34 is selected as the backbone. The mAP reaches 0.9682, and the speed of detecting a single image can be as low as 0.076 s. Secondly, the trained detector is used to detect the video frames, and images with detected boxes and documents with coordinates can be obtained. The position relationship between the detected work objects and detected workers is used to determine how many workers (***N***) have participated in the task. The time (***T***) to perform the process can be obtained from the change of coordinates of the work object. Finally, the productivity is evaluated according to ***N*** and ***T***. The authors use four actual construction videos for validation, and the results show that the productivity evaluation is generally consistent with the actual conditions. The contribution of this research to construction management is twofold: On the one hand, without affecting the normal behavior of workers, a connection between construction individuals and work object is established, and the work productivity evaluation is realized. On the other hand, the proposed method has a positive effect on improving the efficiency of construction management.

## 1. Introduction

Construction sites usually consist of various elements such as personnel, machinery, materials, method, and environment. At the same time, construction sites are also characterized by multiple work types, simultaneous operation of various large-scale pieces of machinery and complex construction environments. Any problem in any links mentioned above may lead to hidden construction quality problems and threaten structural safety and personal safety. Therefore, it is necessary to comprehensively supervise construction sites’ condition in real-time [1]. Work productivity is an essential factor in engineering construction, which directly affects each process’ completion time and quality. However, limited by human resources and time costs, most construction parties only control the project’s progress at a macro level. They do not supervise and record the work productivity of each process in detail.

In the field of engineering construction, most traditional monitoring methods are based on manual monitoring. However, manual supervision often fails to cover every aspect, preventing complete and timely notification of hazardous conditions, irresponsible and irregular worker behavior, sub-standard work quality, and inefficient task execution at construction sites. Therefore, more supervisors are needed, which increases management costs. As a result, many advanced technologies have been introduced into engineering construction, such as the application of acceleration sensors for structural health monitoring [2,3,4] and workers’ activity recognition [5,6,7,8]. However, contact sensors may cause some inconvenience to construction when performing practical applications in engineering. Therefore, an efficient way is to use a non-contact monitoring sensor with high accuracy using video and image signals. In recent years, artificial intelligence and computer vision have accelerated, showing new features such as deep learning, cross-border integration, human–machine collaboration, the openness of group intelligence, and autonomous control [9]. Related deep learning-based monitoring methods have been applied to medical diagnosis [10,11,12], food inspection [13,14,15], vehicle identification [16,17,18], and structural health monitoring [19,20,21,22], providing the possibility to solve problems related to engineering construction.

**Figure 1:**
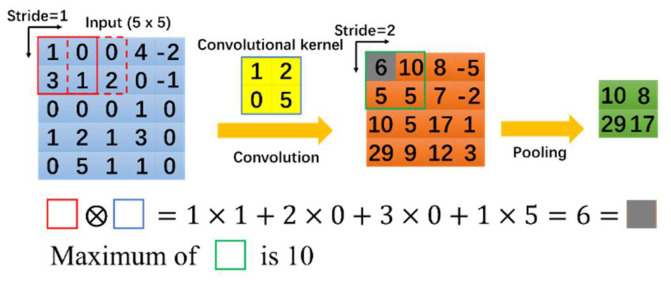
Convolution and pooling operations.

The convolutional neural network (CNN) is the most common deep learning network, which originated from the handwritten numbers recognition problem proposed by Lecun in the 1990s [23]. By optimizing the configuration of convolution and pooling layers, many CNNs have been developed, including AlexNet [24], ZFNet [25], VGGNet [26], GoogleNet [27], and ResNet [28]. An effective way to improve CNNs is to increase the number of layers. In this way, the approximate structure of the objective function can be obtained using the increased nonlinearity, and better features can be obtained. Figure 1 illustrates the operation principle of convolution and pooling layers in a CNN. Several object detection methods based on CNNs that can be applied to different scenes have been developed. Currently, there are various object detection algorithms, including CenterNet [29] and Faster R-CNN [30], such as Mask R-CNN [31] and FCN [32] for object segmentation, and YOLO [33,34,35,36], SSD [37] and MobileNet [38,39] for fast detection of mobile devices.

The urgent need to solve practical problems in engineering construction and the growing maturity of deep learning technology have contributed to the rapid development of computer vision technology in engineering construction in recent years. A large amount of literature has focused on this issue.

The first is construction individual-related issues, which receive more attention by recognizing workers’ activities and usage of personal protective equipment (PPE). In activities recognition: Luo and Li et al. [40,41,42] used various computer vision algorithms for construction worker activity recognition; Cai et al. [43] and Liu et al. [44] also carried out computer vision-based approaches for construction activities’ recognition. Cai used a two-step LSTM (long short-term memory network), while Liu combined computer vision and natural language processing methods; Han et al. [45], Yu et al. [46] and Yang et al. [47] extracted workers’ joint coordinate to recognize the activity and judge the safety status. In the aspect of PPE’s usage: Park et al. [48], Fang et al. [49], and Wu et al. [50] introduced different computer vision-based methods for hardhat detection; Fang et al. [51] and Tang et al. [52] achieved PPE usage detection not limited to hardhats.

The second is material-related issues, Zhang and Zhao et al. [53,54] presented a bolt looseness detection method based on MobileNet. Cha et al. [55,56,57,58] used deep learning technology based on convolutional neural networks to complete the identification and location of surface cracks in concrete structures, the volume measurement of surface corrosion on steel structures, and the volume measurement of concrete spalling damage. Concrete surface defect identification is also an issue that has been studied frequently in recent years, and representative ones are Xu et al. [59], G Li et al. [60], S Li et al. [61], Miao et al. [62] and others. However, most objects covered in the literature mentioned above are materials of existing built structures, which are not strictly speaking construction materials. These studies are more focused on damage detection of building structures. In the aspect of construction materials, Li et al. [63] proposed a YOLOv3-based method for counting rebars. He et al. [64] introduced an object detection framework called Classification Priority Network for defect detection of hot-rolled steels. Zhou et al. [65] described an approach to analyze concrete pore structure based on deep learning.

The third is machinery-related issues, Kim et al. [66] used unmanned aerial vehicles and monitored mobile machinery devices on a construction site remotely based on the YOLOv3 algorithm. Roberts et al. [67] combined unmanned aerial vehicles and image recognition technology to track a crane on a construction site and estimated the three-dimensional posture. Slaton et al. [68] introduced a method to recognize activities of roller compactor and activator by using a convolutional recurrent network. Yang et al. [69] proposed a video monitoring method to evaluate the working state of a tower crane. Yang et al. [70] successfully identified the safe distance between the hook and the worker using a monitoring camera installed on a tower crane.

These studies have contributed to taking a significant step forward in introducing computer vision technologies to construction engineering. However, there are still some limitations:

- In the case of individuals and machinery, most extant computer vision-based approaches focus only on safety monitoring and activity recognition. No literature has been found to use computer vision to analyze their work productivity in some dynamic processes.
- In terms of material, no studies have focused on the changes in materials during dynamic construction, and no studies have connected them to individual work and work productivity.
- Although we have not found studies addressing work productivity evaluation, the large number of successful applications of deep learning and computer vision in engineering construction illustrate their potential to assist in filling this research gap in work productivity evaluation.

When evaluating work productivity, it is necessary to know the time consumed and the number of workers involved in the task, and it will take much time to use manual methods to make statistics. To address the above issues, the authors select construction processes of assembling column reinforcement (ACR) and assembling beam reinforcement (ABR) as research cases and propose a new computer vision-based method for work productivity evaluation. Firstly, we train a detector that can accurately distinguish various assembling reinforcement-related entities using the video images collected from on-site surveillance cameras. An anchor-free center point estimation network (CenterNet) is adopted, which can have a good detection speed without loss of accuracy. Secondly, we determine the number of workers who participated in the ACR\ABR task in every moment according to the position relationship between the detected work object and the detected workers to establish a connection between the workers and construction materials. Finally, we record the change of coordinates of the work object in the video and evaluate the work productivity by combining the time consumed and the number of participants. In this article, computer vision technology is used to realize construction activity recognition of ACR\ABR work, and the number of workers participating in the task can be judged. Additionally, using the results output by CenterNet, the productivity evaluation of the ACR\ABR process is realized. Final inspection documents, tables, and work productivity images can be used for project managers to view the work details of this process more intuitively and quickly. The rest of this paper is organized as follows. Section 2 describes the proposed method in detail. Section 3 describes the establishment of the CenterNet model. Section 4 reports the evaluation tests based on construction video clips. Section 5 is the comparison. Section 6 and Section 7 outline the discussion of the results and conclusions, respectively.

## 2. Methods

### 2.1. Preparation of Dataset

**Figure 2:**
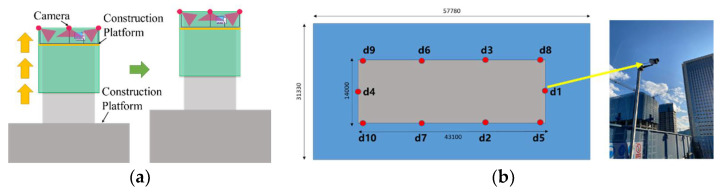
Detail of the construction project: (**a**) Front elevation diagram of slip-form construction; (**b**) Camera layout.

In this paper, images used to train the CenterNet model are from the construction site of a super high-rise project in Donggang Business District, Dalian City, Liaoning Province, China. The structural type of the project is frame–core wall structure, and the slip-form construction technology, as shown in Figure 2a, is applied. With the increase in the storey, the fixed surveillance cameras installed on the construction site will not be able to record the conditions of the construction platform. Therefore, according to Figure 2b, in this research, several cameras that can continuously record construction videos without being disturbed by the increase in the storey were arranged at the edge guardrail.

**Figure 3:**
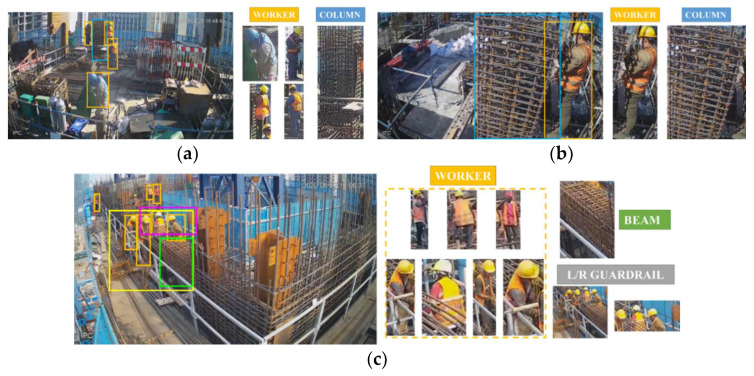
Detail of the dataset: (**a**) Image from D02, (**b**) Image from D06, (**c**) Image from D09.

The cameras selected are all Dahua brand, with 4.0 megapixels and a focal length of 4 mm. All cameras were connected to a switch through network cables, and then the switch transmitted video signals to a PC. The whole video recording system was equipped with a special power supply module to ensure continuous video recording. Four construction video clips from cameras No. 2, No. 6, and No. 9 were extracted. In the videos, workers assemble beam reinforcements (ABR) and assemble column reinforcements (ACR). The behavior studied in this paper is the installation and binding of stirrups after the longitudinal reinforcement is installed. From the videos, 1051 static images were intercepted. The images were not randomly intercepted to better show the changes in construction materials throughout the process; the beginning, proceeding, and ending phases of the task account for 20%, 60%, and 20% of the dataset, respectively. Table 1 shows the source of images in detail. The dataset contains five categories: workers performing construction tasks (“worker”), parts indicating the assembled reinforcement in beams and columns (“beam” and “column”), and guardrails to assist in determining the location of workers (“Lguardrail” and “Rguardrail”). Figure 3 shows some examples of the dataset.

### 2.2. CenterNet

In the initial years of object detection, anchor-based algorithms [30,31,32,34,35,36,37,38,39] dominated. Anchor boxes are essentially a kind of candidate box, and after designing anchor boxes of different scales, CNNs are trained to have the ability to classify the candidate boxes. Eventually, it can distinguish whether the candidate box contains objects and what objects are contained in it. Among various anchor-based algorithms, the two-stage-based have higher accuracy, but it takes more time and computing power to generate candidate boxes in the prediction stage. A one-stage-based algorithm has a faster speed than a two-stage-based algorithm, but accuracy is often lower. In recent years, some scholars have gradually started to study the anchor-free method, which directly eliminates the step of anchor boxes.

**Figure 4:**
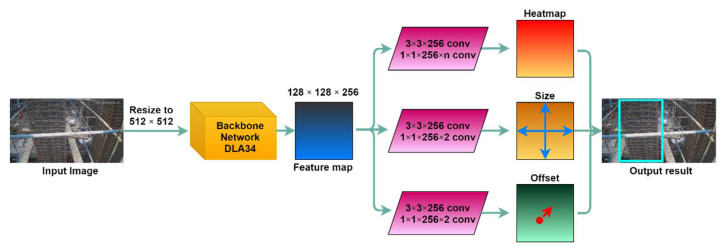
Flowchart of CenterNet.

CornerNet [71] is the first to introduce the method of predicting detected boxes through key points, which first predicts the two corner points of the rectangular box and then regresses the rectangular detected box. CenterNet takes this idea, but the difference is that it regresses detected boxes through the center point. Figure 4 shows the structure of CenterNet. In the prediction stage, input image is resized into 512 × 512 × 3, and the backbone network is used for feature extraction (DLA34 is chosen for the backbone network in this paper). Then, the 128 × 128 × 256 feature map obtained by down-sampling is predicted, and the heatmap, size (***w*** and ***h***), and offset are obtained. The specific way to extract the detected box is to use 3 × 3 max pooling for the heatmap, check whether the value of the current point is larger than the value of the surrounding eight neighboring points, and then filter from the eligible points. Combined with the offset, the center point can be obtained. Finally, coordinates of four corner points of the detected box are calculated from Equation (1) [29]:

[***X_min_***, ***X_max_***, ***Y_min_***, ***Y_max_***] = [(***X_c_*** − ***w***/2), (***X_c_*** + ***w***/2), (***Y_c_*** − ***h***/2), (***Y_c_*** + ***h***/2)] (1)

in which ***w*** is the width of the detected box, and ***h*** is the height of the detected box. ***X_c_*** and ***Y_c_*** are coordinate values of the center point.

In the last two years, some scholars have used this algorithm to solve practical problems, such as pedestrian detection [72], phoneme recognition [73], foreign object detection [74], and ship detection [75], and achieved satisfactory recognition accuracy and speed. Although deep learning is still evolving rapidly, most current research based on object detection still only implements the output of detected boxes, i.e., an account of what class of object is detected in the image. Information embedded in detected boxes is not exploited in-depth, and the three limiting points proposed in the first section are not addressed. Combined with the excellent balance of detection speed and accuracy shown by CenterNet in [72,73,74,75], it is chosen to detect objects related to the ABR\ACR process.

### 2.3. Evaluation for the Productivity

Before evaluation of ACR\ABR productivity, the performance of the model must be guaranteed. We trained several CenterNet-based models and selected the one with the highest mAP.

**Figure 5:**
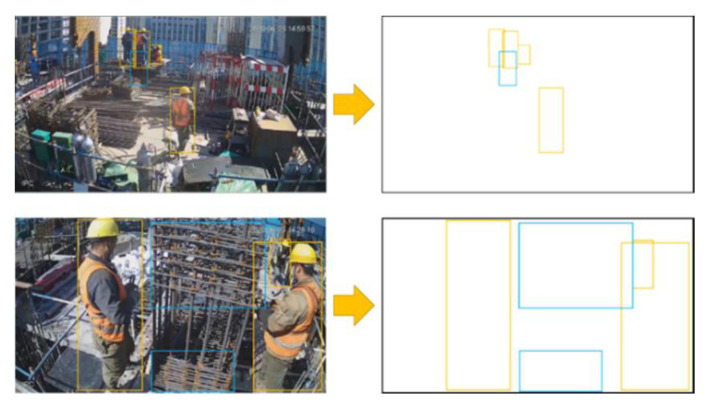
Detected boxes during ACR process.

After identifying the various types of ACR\ABR-related objects, it is necessary to find out the relationship between them according to the position of detected boxes. Figure 5 shows the positional relationship between “worker” and “column” at a specific ACR process moment. It can be seen that three of the four workers in the figure above are performing the ACR process. In the figure below, two of the three workers are performing the ACR process. After verifying 100 images, it was found that the coordinates of workers’ detected boxes performing this process all satisfied the three conditions from Equations (2)–(4).

***Y_worker_min_*** ≥ ***Y_column_min_*** (2)

|***X_worker_max_*** – ***X_worker_min_***| ≥ 0.3|***X_column_max_*** – ***X_column_min_***| (3)

|(***X_worker_max_*** + ***X_worker_min_***)/2 − (***X_column_max_*** + ***X_column_min_***)/2| ≤ 2.5|***X_worker_max_*** − ***X_worker_min_***| (4)

in which ***Y_worker_min_*** and ***Y_column_min_*** represent the minimum and maximum values of the coordinates of “worker’s” detected box and “column’s” detected box in the Y-axis direction, respectively. ***X_worker_max_*** and ***X_worker_min_*** are the maximum and minimum values of the “worker’s” detected box, respectively. ***X_column_max_*** and ***X_column_min_*** are the maximum and minimum values of “column‘s” detected box, respectively.

Equation (2) indicates that the height of the worker in Y-axis direction is not lower than the column when he performs this work, Equation (3) limits the distance of the worker and the column from the camera, and Equation (4) indicates that the worker should surround the processed object.

**Figure 6:**
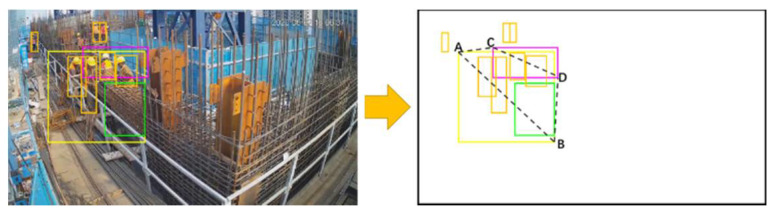
Detected boxes during ABR process.

Figure 6 shows the position of each detection box at a given moment during the ABR process, which contains four categories, namely “Lguardrail”, “Rguardrail”, “beam”, and “worker”. From the coordinates of “Lguardrail” and “Rguardrail”, a quadrilateral ABCD can be obtained. When the worker performs the ABR process, his position is inside the guardrail. The detection image shows the phenomenon presented in Equation (5) that the worker’s detected box intersects the quadrilateral:

***A_ABCD_*** ∩ ***A_worker_*** ≠ 0 (5)

in which ***A_ABCD_*** refers to the area of quadrilateral ABCD. ***A_worker_*** refers to the area of worker detected box.

**Figure 7:**
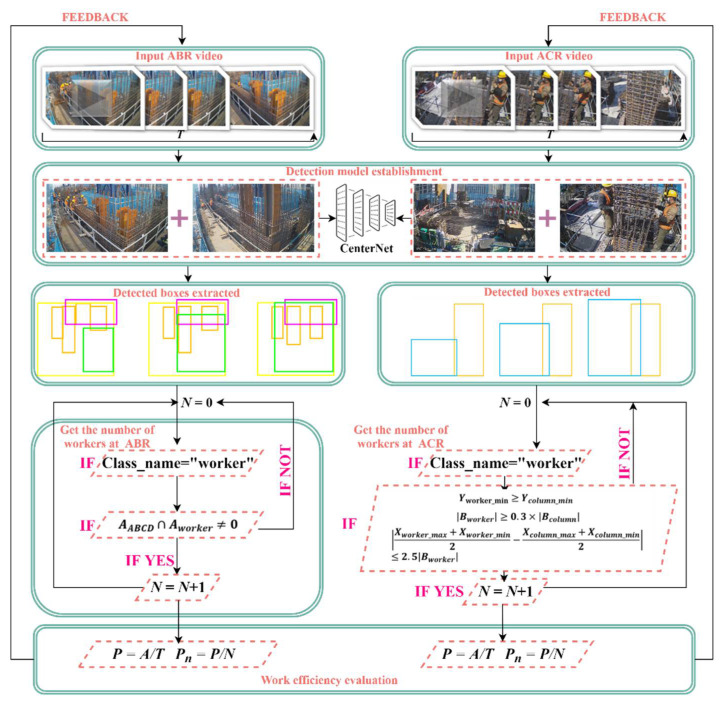
Flowchart of the proposed method.

This article uses the above Equations (2)–(5) as the basis for identifying the ACR\ABR process. After identifying the ACR\ABR process, the work productivity evaluation can be carried out using the obtained detected results. When a worker performs the ACR\ABR process, the number of stirrups will increase in one direction, and the change of coordinates is shown in the detection image. In this paper, the height ***H*** of the “column’s” detected box in the ACR process and the diagonal length ***L*** of the “beam’s” detected box in the ABR process are selected as indicators. When these two values do not change significantly within ten minutes, and no more workers perform the ABR/ACR process, the duration of the process is recorded. The work productivity can be calculated from Equation (6):

***P*** = ***A***/***T***; ***P_n_*** = ***P***/***N*** (6)

in which ***P*** represents the work productivity of the ACR\ABR process. ***P_n_*** is workers’ average work productivity. ***A*** is an amplification factor, which is taken as 10,000 in this paper. ***T*** refers to the duration to perform the task. ***N*** refers to the average number of workers performing the ACR\ABR process. The usual method used to calculate work productivity is to divide workload by work duration. In this study, the workload is the same when performing the same kind of process, so the inverse of ***T*** can be directly considered as the work productivity. However, as a variable representing time, the value of ***T*** may be tremendous. For example, when ***T*** = 5000 s, ***P*** is only 0.0002. Too many digits after the decimal point may be unfavorable for analysis and comparison. Therefore, to better present the evaluation results, the method used in this paper is to multiply by an amplification factor ***A***. By dividing the calculated ***P*** by ***N***, we can obtain the ***P_n_***. Figure 7 shows the flowchart of the proposed method.

## 3. Establishment of CenterNet Model

### 3.1. Operating Environment and Parameter Settings

In this article, a PC with NVIDIA 1080ti GPU, Core i7-8700 CPU, 16GB RAM, and Pytorch framework is applied for training. In the dataset, the ratio of the number of images in the training set to the test set is 8:2. Eighty percent of the images are used to train the model in the training set, and the remaining twenty are used for validation. Different learning rates (0.0001, 0.0005) and different batch sizes (1, 2, 4, 8, and 16) are combined. Ten detection models are trained, from which the best is selected. Epoch is set to 200 uniformly, and evaluation is performed every five epochs.

### 3.2. Training Results

**Figure 8:**
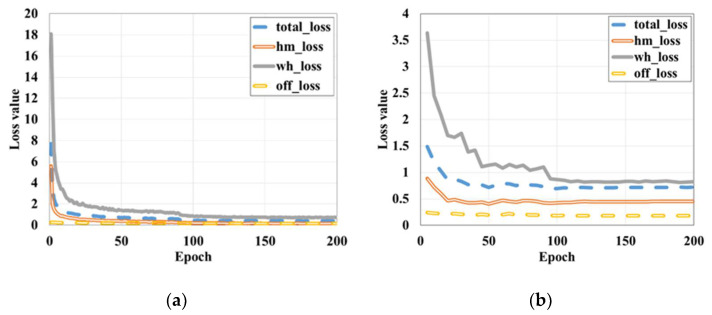
Training and validation losses versus epochs: (**a**) Training loss, (**b**) Validation loss.

**Figure 9:**
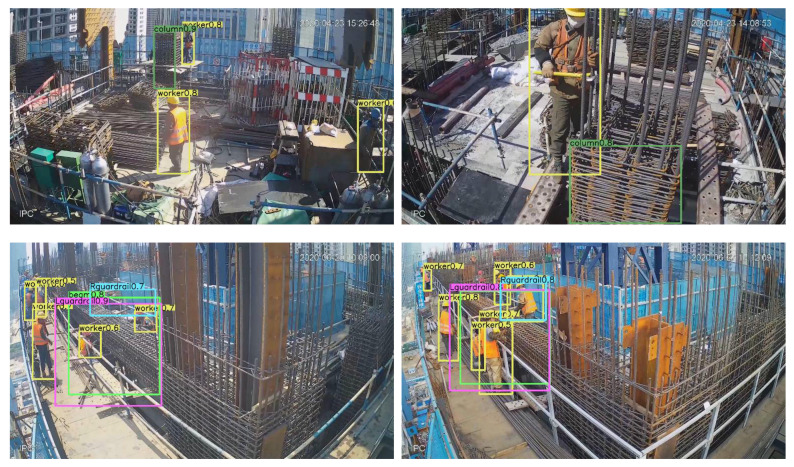
Detection results based on CenterNet.

Table 2 lists the AP (Average Precision) and mAP (mean Average Precision) of the trained models. Among them, AP represents the recognition accuracy of each category, and mAP is the average value of AP, which represents the overall classification performance of the model. It can be seen from Table 2 that the model with a learning rate of 0.0001 and a batch size of 2 has the best recognition ability when it is trained to the 110th epoch, and its mAP is 0.9682. Figure 8a,b show the training loss and validation loss, respectively. Loss value indicates the deviation of the predicted value from the actual value, and the smaller the value, the lower the deviation. As we can see from the curves, the loss value decreases continuously as the training continues, and the validation loss reaches the minimum value at the 110th epoch. Then, it smooths out at the end, indicating that the model has reached convergence. Eventually, this 110th epoch model is retained for subsequent studies. To verify the feasibility of the model trained in this paper, we also applied three classical anchor-based object detection algorithms, the one-stage SSD and YOLO v3, and the two-stage Faster R-CNN. The mAP values and the time consumed (***t***) to detect a single image are listed in Table 3. It can be seen that the model trained in this paper is 3.125 times faster than the Faster R-CNN, although it is slightly inferior to the Faster R-CNN in terms of accuracy. Compared with YOLO v3, the recognition speed is slower, but the accuracy is higher. In other words, the CenterNet-based detection model has a good balance of speed and accuracy. From the partial detection results listed in Figure 9, each object can be well recognized in the images. Therefore, it is feasible to select this object detection model for the subsequent work productivity evaluation.

## 4. Experiment

### 4.1. ACR\ABR Activity Recognition

**Figure 10:**
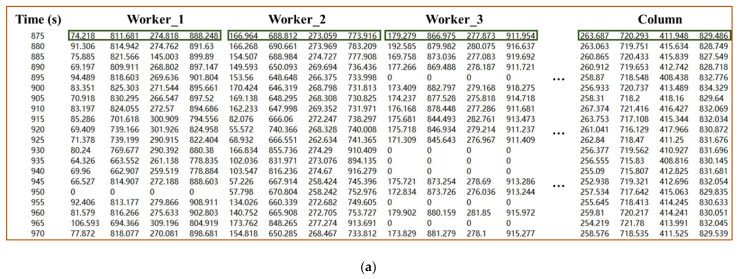

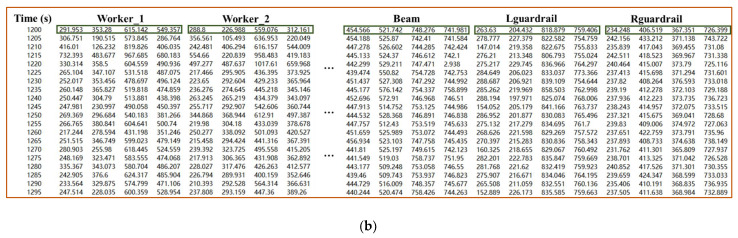
Coordinates of boxes at each moment: (**a**) ACR, (**b**) ABR.

After detection results are obtained, the next step is to use the detected box of each object to identify ABR and ACR’s activity through Equations (2)–(5) and then determine how many workers have performed these two tasks. To test the method’s practical effectiveness, videos described in Section 2.1 are input into the trained CenterNet model, and Table 4 lists the information such as the duration of these videos. The frame rate is 25, which means that there are 25 images per second. If the detection model is used to detect all 25 images per second, it will increase the computer memory consumption. Therefore, the video is detected every five seconds (125 frames) after input into the CenterNet model. Finally, after processing, the detection document is generated, as shown in Figure 10. Inside the document, the category detected at the current moment and the coordinates of detected boxes are recorded in detail, and the four values represent the ***Y_min_***, ***X_min_***, ***Y_max_***, ***X_max_***, respectively.

After obtaining the coordinate values shown in Figure 10, the first step is to distinguish which process this video belongs to. When Δ***L*** > Δ***H***, an ABR process is executed in the video. When Δ***L*** < Δ***H***, that is an ACR process. Δ***L*** and Δ***H*** are calculated as shown in Equation (7):

Δ***L*** = ***L_end_*** − ***L_start_***; Δ***H*** = ***H_end_*** − ***H_start_*** (7)

in which ***L_end_*** and ***L_start_*** refer to the length of the diagonal line of the “beam’s” detected box at the end of the task and at the beginning of the task, respectively. ***H_end_*** and ***H_start_*** represent the height of the “column’s” detected box at the end of the process and the height at the beginning, respectively. Since ABR and ACR work will not be performed simultaneously during the construction of the building structure in this paper, Δ***H*** is usually 0 when performing ABR tasks, and Δ***L*** is usually 0 when performing ACR tasks.

For “worker’s” coordinates at each moment in the document, ACR\ABR activity recognition is performed according to the flow in Figure 7. For the number of workers ***N*** participating in the ABR/ACR task at each moment, an initial value ***N*** = 0 is assigned. Equation (5) is executed for the coordinates of each “worker” in the ABR task, and ***N*** is added by 1 when a worker is detected satisfying the condition. For each “worker’s” coordinate in the ACR task, we carried out the Equations (2)–(4). ***N*** is added to 1 for each time when the condition is satisfied at this moment. By iterating through each line of the detection document in this way, the number of workers performing the ACR\ABR process at each moment is obtained.

**Figure 11:**
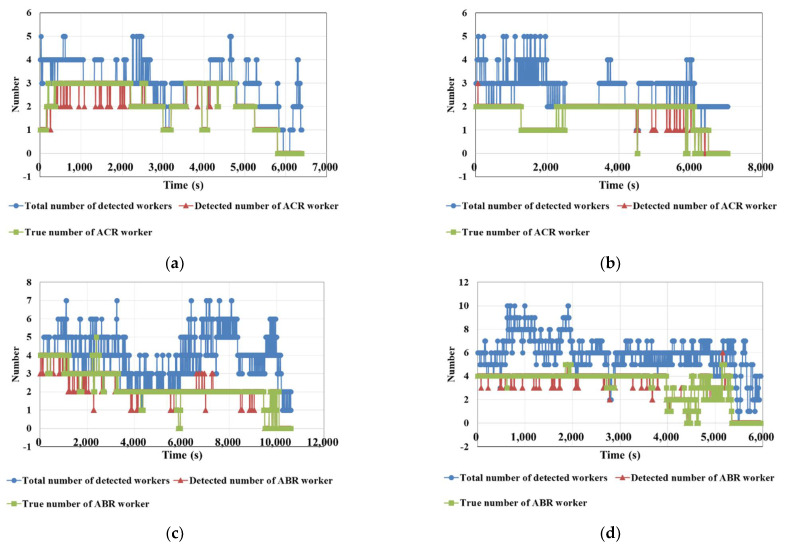
Curves of the number of detected workers: (**a**) V01, (**b**) V02, (**c**) V03, (**d**) V04.

The authors counted the total number of detected workers at each moment, the number ***N_detected_*** of workers involved in ABR\ACR work detected by the method we proposed in Section 2.3, and the true number ***N_true_*** of workers involved in ABR\ACR work. Figure 11 presents the three values above, and it can be seen that ***N_true_*** and ***N_detected_*** have a good overlap. Table 5 lists the number of moments when the count is correct (***N_true_*** = ***N_detected_***) and the number of moments when the count is incorrect (***N_true_*** ≠ ***N_detected_***). The errors in V01 and V02 are mainly due to workers entering the blind area of the camera, while the errors in V03 and V04 are mainly due to some workers being blocked by other workers and unrelated individuals entering the working area of the ABR.

To test the accuracy of ABR\ACR activity recognition, we calculated ***N_true_total_*** and ***N_detected_total_*** according to Equation (8), and the calculated results are listed in Table 6:

(8) $$N_{true\_total}\;=\;\sum_{t=0}^{t=end}N_{true};\;N_{detected\_total}\;=\;\sum_{t=0}^{t=end}N_{detected}$$

The ***N_true_total_*** and ***N_detected_total_*** in Equation (8) refer to the sum of ***N_true_*** and ***N_detected_*** for each moment from the start to the end of the process, respectively. From the results in Table 5 and Table 6, it can be seen that the moments in which the number of workers involved in the ABR\ACR process is correctly accounted for in 93.4% of all moments, and the average accuracy of ABR\ACR’s activity recognition reached 0.970. The errors are mainly caused by workers being completely obscured and irrelevant workers staying in the work area.

### 4.2. Evaluation for Work Productivity

**Figure 12:**
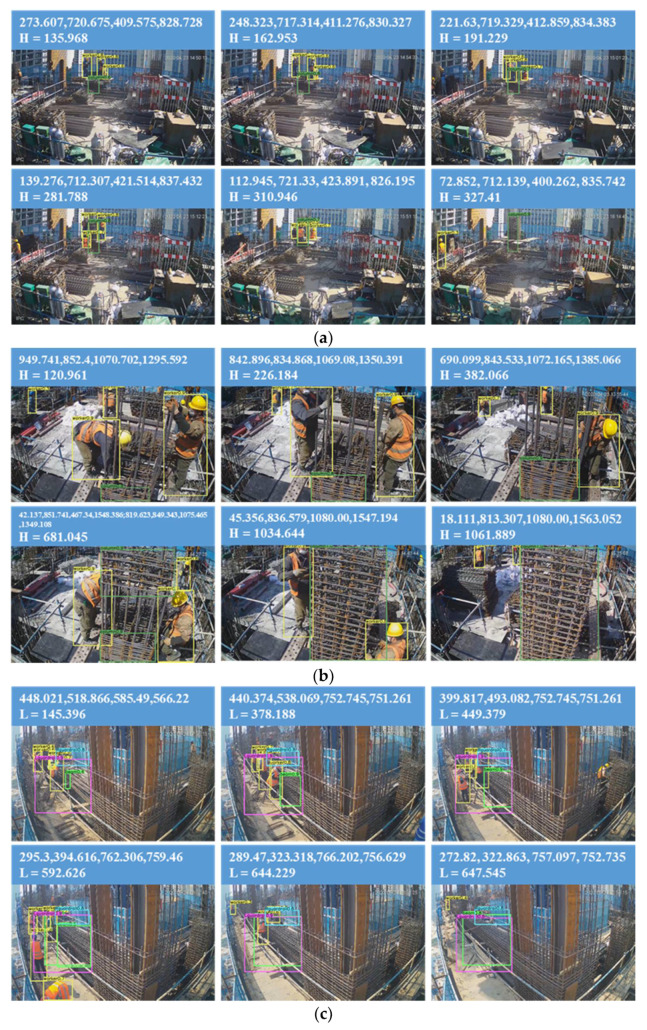

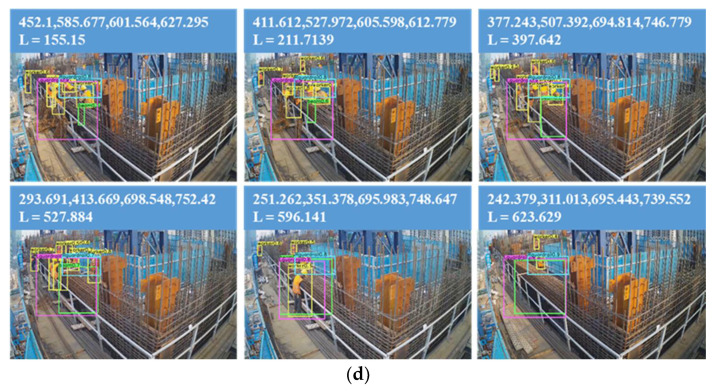
Test images of the four videos: (**a**) V01, (**b**) V02, (**c**) V03, (**d**) V04.

Figure 12 shows some of the images when the CenterNet-based model is used to detect these videos. The trained detection model accurately reflects the change in the coordinates of the working object during the ACR\ABR process, and both the ***H*** and ***L*** values gradually increase with the work.

**Figure 13:**
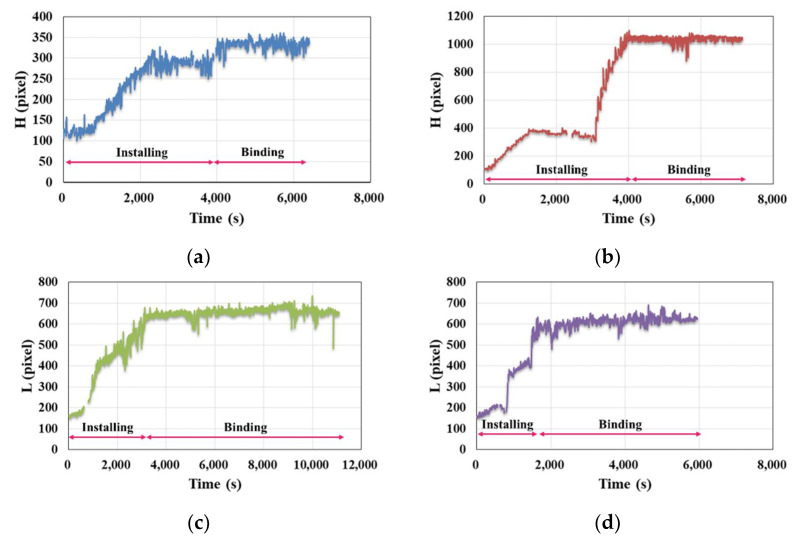
Curves of the H\L versus time: (**a**) V01, (**b**) V02, (**c**) V03, (**d**) V04.

To further illustrate this phenomenon, the coordinates of “column” and “beam” are extracted from the detection file shown in Figure 10, and the curves of these two values versus time shown in Figure 13 are plotted. Although there are fluctuations in the curve due to errors, the trend accurately reflects the actual condition of the ACR\ABR construction process. The information contained is sufficient to assist in evaluating work productivity. It also shows, from the side, that the model trained by applying the dataset prepared in Section 2.1 can accurately reflect the changing trend of construction materials. As shown in Figure 13, the process can be divided into two stages: first is the Installing stage, in which the workers assemble all the stirrups into the final finished shape. Then, in the Binding stage, the workers use thin iron wires to fix the stirrups to the longitudinal bars. In Figure 13a,b, there is a phenomenon that the ***H*** value first rises and then falls in the first stage. It is caused by the fact that workers push them down after the stirrups are assembled to a certain height, and the spacing between them becomes smaller. After the longitudinal force reinforcement is connected, the assembly work is resumed until it is formed.

**Figure 14:**
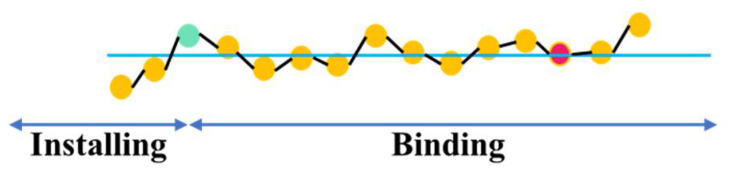
Determination of the dividing point between “installing” and “binding”.

To determine ***T*** for each stage, the method shown in Figure 14 is adopted, i.e., the point with the smallest value among the last five points is selected, and then a line with slope 0 is made to the left. The point to the right of the last intersection is taken as the dividing point between Installing and Binding.

From the data in Figure 11, the average number of workers involved in each stage of the ABR\ACR process can be calculated, and from Figure 13 and Figure 14, ***T*** for each stage can be obtained, and from these conditions, ***P*** and ***P_n_*** can then be calculated from Equation (6). The final results are shown in Table 7.

When calculating ***P*** and ***P_n_*** in Table 7, a uniform ***A***-value is used as the ***T_installing_*** is different from ***T_binding_***, which leads to a significant difference in ***P*** and ***P_n_*** between different stages of the same ABR\ACR process. The workload between different stages is different, so the ***P*** and ***P_n_*** in Table 7 are not applicable for vertical comparison but can only be used for horizontal comparison. It can be seen that the ***P*** of V01 is 11.3% higher than that of V02. It is because its ***N*** is also greater than V02, which is 32.9%. The advantage of V01’s ***P*** compared to V02 is not as big as the difference between ***N***. This is also reflected in ***P_n_***’s value, which is 16.3% lower for V01 than V02. When viewed in stages, in the installing stage, the ***P_installing_*** of V01 is slightly higher than that of V02. However, the difference in ***N_installing_*** between the two is as high as 0.71, so the ***P_n_installing_*** of V01 is lower than V02. In the binding stage, the ***P_binding_*** of V01 is 21.5% higher than that of V02. However, the ***N_binding_*** is only 14.1% higher than V02, which indicates that the ***P_n_binding_*** of V01 should be higher than that of V02. The data in Table 7 confirm this. In comparing V03 and V04, the ABR process of the beam in the same position, V04’s productivity in all stages is higher than V03’s, and with a slight difference in ***P_n_***, it can be seen that ***N*** plays a key factor.

Combined with Table 7 and Figure 13, it can be seen that the productivity evaluation index calculated by the coordinate detected by CenterNet conforms to the actual situation and satisfies the objective law. It is worth mentioning that the dataset of this paper has 1051 images. As many as 6239 images are involved in CenterNet detection in this section, indicating that the proposed computer vision method has relatively good stability. The results obtained in this paper validate that using a computer vision-based method to assist in productivity evaluation is a feasible approach, and the generated images help assist construction managers to analyze better the reasons for the excessively fast or slow construction speed, which in turn helps them to allocate on-site human resources and improve construction productivity rationally.

## 5. Comparison

To further verify the robustness of the CenterNet-based deep learning model used in this paper, Table 8 explores the evaluation of the productivity of the ACR process after replacing the object detection algorithm with other algorithms, using V02 as a case study. The actual values are listed for comparison.

The data listed in Table 8 show that applying the work productivity evaluation method we proposed in Section 2.3 in combination with different object detection algorithms can achieve good results. Even the SSD algorithm with lower precision has a maximum error of only 8% compared with the actual value. It shows that the work productivity evaluation with the method proposed in Section 2.3 is feasible and further verifies the effectiveness of the object detection algorithm in solving the productivity evaluation problem in engineering construction. Comparing the four object detection algorithms together, CenterNet and Faster R-CNN have better agreement with the actual results, mainly due to “worker’s” lower missed detection rate. Combined with the advantages of CenterNet in detection speed, the CenterNet-based object detection model can be considered comparable and applicable to the problem presented in this paper.

## 6. Discussion

In the field of engineering construction, construction productivity has not received much attention for a long time. Generally, as long as the tasks that should be carried out are completed by the specified deadline, most construction companies do not add the cost of monitoring each process’ speed. Noting the potential of computer vision technology for applications in engineering construction, the authors propose a computer vision-based approach for the productivity evaluation of assembling reinforcement processes. This paper contributes to engineering construction in the following aspects:

Firstly, advanced deep learning technology is employed to detect the frequency observed five classes of objects in ABR\ACR images. To achieve this plan, the authors collect and annotate the dataset to train the CenterNet model and evaluate the performance through the test set. It is found that the CenterNet-based model presents satisfactory mAP and detection speed.

Secondly, based on the detected ABR\ACR-related objects, a connection between the worker and construction object is established. The ABR\ACR task can be recognized through the position relationship between the worker and the construction object, so as to obtain the number of workers who participate in the process (***N***). The time (***T***) to perform the task can also be obtained through the recognized materials changing.

Thirdly, with ***N*** and ***T***, productivity can be evaluated. The results of this paper validate the feasibility of computer vision-based methods in evaluating work productivity. With this paper’s results, managers can check the work productivity in detail, determine which workers are performing inefficient work, and then allocate labor resources more reasonably to promote and improve the complete quality of the whole project, forming a virtuous cycle. With the refinement of the proposed method and the expansion of its application, the computer vision-based approach will make it possible to perform rapid productivity evaluation for each process in construction.

The study in this paper contains some limitations that need to be further improved in future work. First of all, error analysis: the results of this paper have some errors, and most of these errors are caused by workers or construction objects being obscured. It is a common problem faced by most current computer vision-based methods. When a worker is not completely obscured, it is still possible for him/her to be recognized, for example, at some moments when the worker only shows half of his/her upper body, but it can still be detected. However, if the worker is completely obscured, it will lead to an error in the judgment of ***N***. When the work object is obscured, it will affect the results of ***L*** and ***H***, and thus fluctuate in the curve shown in Figure 13. In the future, we can consider adding several cameras in a process scene to expand the dataset, to try to reduce errors from multi-view monitoring. Second is the applicability issue: the results of this paper can be extended to other processes of civil engineering construction. Processes such as earthwork, concrete pouring projects, masonry structure projects can apply the ideas of this paper for productivity evaluation, which is the direction in which future work needs to be improved.

## 7. Conclusions

This paper introduces a new method to evaluate the productivity of assembling reinforcement through the position relationship of objects detected by CenterNet. Firstly, a dataset of 1051 images with five categories is created based on entities related to assembling reinforcement. Eighty percent of the dataset is used to train and evaluate the CenterNet model, and the remaining twenty is used to test the detector’s performance. The results showed that the mAP reached 0.9682. Compared with the other three object detection models, the detector trained in this paper is comparable. Then, by inputting the videos into the model, the coordinate of the detected boxes at each moment can be obtained, and the number of workers engaged in the task can be judged through the boxes’ position relationship. Finally, evaluation of work productivity is realized by the change of coordinates of the work object in the video, the time consumed to perform the task, and the number of workers involved in the process.

The work productivity evaluation value obtained matches the construction site’s actual condition and satisfies the objective law, indicating that the application of computer vision to evaluate engineering work productivity is a feasible approach. Applying the dataset proposed in this paper, the trend of construction material changes can be accurately reflected. With this method, project managers can quickly visualize the productivity of assembling reinforcement without a significant cost increase. The information obtained can be used to allocate human resources to construction sites more rationally. As the method is improved, it will potentially to be used for productivity evaluation of various processes in engineering construction.

## Figures and Tables

**Table 1:** Details of the dataset.

Camera Number	D02	D06	D09	D09
data	23 April 2020	23 April 2020	20 June 2020	27 June 2020
task	ACR	ACR	ABR	ABR
number of images	227	286	265	273

**Table 2:** AP and mAP corresponding to each category.

Learning Rate	Batch Size	AP					Best Epoch	mAP
		Worker	Column	Beam	Lguardrail	Rguardrail		
0.0001	1	0.933	0.894	0.869	0.946	0.959	125	0.9202
0.0005	1	0.766	0.936	0.773	0.965	0.923	115	0.8726
0.0001	2	0.935	0.973	0.943	0.996	0.980	110	0.9682
0.0005	2	0.952	0.957	0.869	0.996	0.991	135	0.953
0.0001	4	0.947	0.979	0.942	0.996	0.955	95	0.9638
0.0005	4	0.933	0.975	0.922	0.998	0.959	125	0.9574
0.0001	8	0.943	0.965	0.951	0.997	0.965	100	0.9642
0.0005	8	0.943	0.969	0.929	0.996	0.941	125	0.9556
0.0001	16	0.936	0.966	0.910	0.995	0.976	100	0.9566
0.0005	16	0.948	0.984	0.927	0.996	0.965	100	0.9551

**Table 3:** Comparison between different object detection algorithms.

	mAP	t (s)
Centernet	0.9682	0.076
Faster R-CNN	0.9751	0.240
SSD	0.9282	0.085
YOLO v3	0.9530	0.060

**Table 4:** Details of the videos.

Video Number	V01	V02	V03	V04
duration	1 h 46 min 30 s	1 h 58 min 50 s	3 h 04 min 30 s	1 h 39 min 05 s
task	ACR	ACR	ABR	ABR
camera	D02	D06	D09	D09
format	MP4			
frame rate	25 Fps			

**Table 5:** Correct moments and incorrect moments.

Video Number	V01	V02	V03	V04
correct	1203	1426	2095	1098
incorrect	75	81	147	94
accuracy	0.941	0.946	0.934	0.921

**Table 6:** Number of detected workers who participate in ABR\ACR process.

Video Number	V01	V02	V03	V04
***N_true_total_***	2721	2257	4671	3885
***N_detected_total_***	2629	2176	4560	3799
accuracy	0.966	0.964	0.976	0.977

**Table 7:** Evaluation of work productivity.

Video Number	V01	V02	V03	V04
***T*** (s)	6410	7135	11,075	5960
***T_installing_*** (s)	3870	4050	3210	1565
***T_binding_*** (s)	2540	3085	7865	4395
***N***	2.06	1.55	2.13	3.19
***N_installing_***	2.41	1.70	3.19	3.95
***N_binding_***	1.53	1.34	1.68	2.93
***P***	1.560	1.402	0.903	1.672
***P_installing_***	2.584	2.470	3.115	6.389
***P_binding_***	3.937	3.241	1.271	2.275
***P_n_***	0.757	0.904	0.424	0.524
***P_n_installing_***	1.072	1.453	0.976	1.617
***P_n_binding_***	2.57	2.419	0.757	0.776

**Table 8:** Comparison when evaluating the work productivity of V02.

	Real	CenterNet	Faster R-CNN	SSD	YOLO v3
***T*** (s)	7135	7135	7135	7135	7135
***T_installing_*** (s)	4060	4050	4045	4035	4050
***T_binding_*** (s)	3075	3085	3095	3100	3085
***N***	1.59	1.55	1.55	1.52	1.54
***N_installing_***	1.71	1.70	1.70	1.68	1.69
***N_binding_***	1.44	1.34	1.34	1.31	1.33
***P***	1.402	1.402	1.402	1.402	1.402
***P_installing_***	2.463	2.470	2.472	2.478	2.470
***P_binding_***	3.252	3.241	3.231	3.225	3.241
***P_n_***	0.882	0.904	0.904	0.922	0.910
***P_n_installing_***	1.440	1.453	1.459	1.475	1.461
***P_n_binding_***	2.268	2.389	2.399	2.461	2.436

## Data Availability

Not applicable.
